# Appetite and Satiety Control—Contribution of Gut Mechanisms

**DOI:** 10.3390/nu13103635

**Published:** 2021-10-17

**Authors:** Christine Feinle-Bisset, Michael Horowitz

**Affiliations:** 1 Adelaide Medical School, Faculty of Health and Medical Sciences, University of Adelaide, Adelaide, SA 5000, Australia; michael.horowitz@adelaide.edu.au; 2 Centre of Research Excellence in Translating Nutritional Science to Good Health, University of Adelaide, Adelaide, SA 5000, Australia; 3 Endocrine & Metabolic Unit, Royal Adelaide Hospital, Adelaide, SA 5000, Australia

The prevalence of obesity, and its comorbidities, particularly type 2 diabetes, cardiovascular and hepatic disease and certain cancers, continues to rise at an alarming rate worldwide [1,2]. This reflects, in part, the suboptimal therapeutic options available for both the prevention and management of obesity. Paradoxically, despite an increasingly obesogenic environment, particularly in Western societies, undernutrition is also extremely common and associated with major adverse consequences for quality of life and healthcare utilisation [3,4]. Older individuals, in particular, may be predisposed to malnutrition as a result of the ‘physiological’ anorexia of ageing. Accordingly, an improved understanding of the mechanisms which regulate appetite and energy intake is of pivotal importance.

Appetite and energy intake are modulated by a diverse range of factors—the latter are both ‘central’ and ‘peripheral’ [5,6]. The regulation of energy intake has been, and continues to be, investigated in both animal and human studies. The focus of animal studies has often been to define mechanisms, with the implicit caveat that findings may not be directly applicable to humans. Moreover, subjective perceptions of appetite cannot be evaluated in animals. In contrast, studies in humans have traditionally addressed phenomenology, as invasive mechanistic studies are not feasible. The application of novel, sophisticated techniques, particularly related to imaging as well as molecular biology, has substantially advanced our understanding of the peripheral and central components of the mechanisms controlling appetite and energy intake in humans. This has led to a redefinition of many concepts, including the relative importance of central versus peri-pheral mechanisms, recognising that the gastrointestinal tract, particularly gut hormones, plays a critical role [7].

During meal ingestion, the oral cavity is exposed to the texture, taste and smell of the food [8]. As food enters the stomach, it is mixed with gastric secretions to produce chyme and progressively distends the stomach to induce a feeling of fullness. As gastric emptying progresses, specialised receptors, located on enteroendocrine cells, are activated by intestinal contents, including nutrients, nutrient digestion products and metabolites, bile acids and other food components, such as bitter substances, triggering the release of gut hormones [9,10]. The latter, in turn, modulate the motor functions of the stomach to further regulate gastric emptying and also activate specialised receptors on vagal afferents to transmit meal-related signals to the brain, leading to meal termination and modulation of reward perception [7]. In recent years, there has been substantial interest in characte-rising the contribution of the microbiota to the regulation of ingestive behaviour. It is now clear that a number of eating-related disorders, including obesity, are associated with dysregulations of gastrointestinal signalling involved in the regulation of eating [7].

Given the major advance in knowledge in the field, this Special Issue is timely, providing a comprehensive overview of the gastrointestinal mechanisms underlying the regulation of appetite and energy intake, as a series of definitive reviews by international authorities. These reviews address gut-related mechanisms, including nutrient sensing, gut hormones, gastrointestinal motility, gut-brain communication and the roles of the vagus, diet and the microbiota, as well as the abnormalities associated with eating disorders, specifically obesity and the anorexia of ageing. The reviews are divided into two sections; the first focuses on preclinical research, and the second on knowledge derived from studies in humans, including the implications for the management of metabolic diseases and eating-related disorders. The reviews are complemented by a number of important original papers.

Lu and colleagues [11] discuss the cellular mechanisms that underlie the secretion of gut hormones released in response to dietary macronutrients and their digestion products, with a focus on the ‘incretin’ hormones, glucagon-like peptide-1 (GLP-1) and glucose-dependent insulinotropic polypeptide (GIP). It is now appreciated that the nutrient-induced release of gut hormones involves a range of mechanisms. An improved understanding of these mechanisms is likely to lead to the identification of novel targets for the management of disordered eating. Page summarises current knowledge about the transmission of gastrointestinal luminal, meal-related signals to the brain via the vagus nerve, the modulatory role of circadian rhythms on vagal responsiveness to luminal signals and the influences of obesity and light cycle disruptions—the latter simulating shift-work [12]. Malbert addresses vagally mediated gut–brain relationships, focusing on important insights derived from studies in pigs [13], particularly in relation to the sensing of information from the stomach, intestines and portal vein by vagal afferents and the central processing of this information, as evaluated by sophisticated imaging techniques. DiPatrizio summarises the evidence that supports an important modulatory role for endocannabinoids in transmitting signals along the gut-brain axis via direct and indirect interactions with vagal afferents [14]. Endocannabinoids activate cannabinoid receptors on vagal afferents directly and also indirectly by mediating nutrient-induced gut hormone secretion from enteroendocrine cells. It is increasingly appreciated that disturbances in the composition of the gut microbiota are associated with a number of dysfunctions, including autoimmune, psychiatric, neurological and metabolic disorders—the latter includes obesity and type 2 diabetes. While knowledge relating to the importance of the microbiota in the regulation of energy intake is still in its infancy, there is increasing evidence that diet-induced changes in the composition of the microbiota impact both eating behaviour and body weight. The review by Rautmann and de la Serre summarises the current understanding of the pathways by which the microbiota may modulate eating behaviour, including gut hormone release and signalling along the vagus nerve [15]. The reinforcing properties of food have the capacity to drive decisions based on reward incentives rather than metabolic needs and are, thus, of potential importance to the regulation of food intake. The review by Decarie-Spain and Kanoski [16] addresses the roles of the gut hormones, ghrelin and GLP-1, in modulating food reward-motivated behaviours, with a particular focus on the opposing effects of the two hormones on a number of behavioural constructs related to food reward and reinforcement. The sensory information from the GI tract enters the central nervous system via the nucleus of the solitary tract, where it is integrated with inputs from other brainstem, midbrain and forebrain nuclei. This information is then transmitted to the dorsal motor nucleus of the vagus, from where feedback signals to the periphery are coordinated. Browning and Carson [17] provide an insightful overview of these circuits and the key centres involved in the regulation of gastric functions, with relevance to the regulation of food intake.

The subjective sensory experience of food, including its appearance, smell, taste and texture, is recognised to play an important role in the regulation of energy intake. Lasschuijt and colleagues [18] discuss the contributions of orosensory factors during food ingestion and mastication, including the palatability and texture of foods, on appetite and energy intake, as well as the central neurophysiological mechanisms that mediate the responses to orosensory exposure. Food ingestion is also associated with and driven by a hedonic experience, including a sense of satisfaction and changes in mood, which are fundamental to a positive postprandial experience. Livovski and Azpiroz review the relationship of this experience with the sensory inputs provided by physiological stimuli that are elicited in the process of meal consumption [19].

The composition of a meal is detected in the oral cavity and throughout the lumen of the gastrointestinal tract. In addition to the four basic taste qualities, sweet, sour, salty and bitter, there is now evidence for the existence of additional tastes, including fat, umami, kokumi and carbohydrate. Keast and colleagues provide an overview of these tastes, their sensing in the oral cavity and gastrointestinal lumen, and their implications for food consumption [20]. As a receptacle and storage organ for food, often for many hours post-consumption, the stomach plays a critical role in the acute regulation of food intake. Distension of the stomach by a meal induces fullness, providing a satiation signal, and gastric emptying regulates the delivery of nutrients to the small intestine triggering gut hormone secretion and nutrient absorption. Cifuentes and colleagues discuss the motor and sensory functions of the stomach, including their assessment using state of the art techniques, the relationship between gastric functions and the regulation of energy intake and the dysfunctions in obesity and consequent implications for effective management [21]. Once dietary nutrients are present in the small intestinal lumen, their effects appear to be region-dependent, with early studies demonstrating that the administration of nutrients directly into the distal small intestine (ileum) potently slows gastric emptying, a phenomenon termed the ‘ileal brake’. It is now recognised that similar ‘intestinal brakes’ exist for the effects of nutrients to reduce appetite and energy intake. Wilbrink and colleagues summarise current knowledge relating to the effects of nutrients administered into the proximal and distal small intestine on appetite and energy intake [22]. In particular, their review highlights the region-dependency of intestinal nutrient-induced modulation of appetite. Bitter substances have recently attracted substantial interest, subsequent to the recognition of their capacity to stimulate gastrointestinal functions (particularly the release of gut hormones and slowing of gastric emptying) that are integral to the regulation of energy intake. Rezaie and colleagues discuss the mechanisms underlying intestinal bitter sensing, the gastrointestinal effects of bitter substances in both preclinical and clinical settings, whether current evidence is indicative of potent energy intake-suppressant effects of bitter substances and the potential clinical implications for the management of obesity [23]. The important contribution of bile acids to a number of gastrointestinal and metabolic functions, including the secretion of gut hormones, is increasingly appreciated. Xie and colleagues summarise the role of bile acids, previously regarded as ‘detergents’, in the regulation of energy intake and body weight, with a focus on the secretion of gut hormones and the changes in obesity and type 2 diabetes [24]. It is clear that bile acids are important signalling molecules, although their precise contribution to the regulation of appetite and energy intake remains to be determined.

Obesity is characterised by dysregulations in the mechanisms controlling energy balance, including energy intake. Farhadipour and Depoortere review the evidence that gut hormone secretion is altered in obesity, reflecting changes in the functionality of gastrointestinal nutrient receptors and the effects of different weight loss strategies [25]. That bariatric surgery is the most effective strategy to achieve long-term weight loss in obesity is likely to be attributable to the markedly enhanced secretion of some gut hormones. Papamargaritis and le Roux discuss current knowledge derived from clinical studies relating to the effects of bariatric surgery on gut hormones and its associated effects to reduce food intake and suppress appetite [26]. In contrast to obesity, which is characterised by relative overconsumption of food, ‘healthy’ ageing is associated with a decline in hunger and the desire to eat, making individuals more susceptible (e.g., as a result of intercurrent illness) to malnutrition and a loss of bodily function, particularly of muscle. Chapman and colleagues summarise the age-related changes in gastrointestinal functions that regulate appetite and energy intake and the implications for the use of dietary supplements, particularly those rich in protein, to cover nutritional requirements and minimise age-related muscle loss [27].

The review articles are accompanied by eight important original articles—the latter attest to the diversity of the work in this field. Preclinical studies investigated the effect of dietary supplementation with the amino acid isoleucine on body weight and blood glucose in lean and obese mice [28]; the effects of obesity on the expression of nutrient receptors and gut hormones in tissue samples of the human colon [29]; the impact of cannabinoid CB1 receptors in the intestinal epithelium on acute dietary preferences for a Western diet in mice [30]; the involvement of specific bitter receptor subtypes, hTAS2R5, hTAS2R14 and hTAS2R39, in the enteroendocrine secretion of CCK, GLP-1 and PYY from intestinal segments in response to a number of bitter agonists; and the relationship with food intake in rats [31]. Clinical studies related to the acute effects of the short-acting GLP-1 receptor agonist lixisenatide (used widely in the management of type 2 diabetes) on energy intake, and the relationship with gastric emptying and intragastric meal distribution, in both health and type 2 diabetes [32]. Other studies investigated the effects of manipulating abdominothoracic postural tone on sensations of digestive ‘well-being’ and bloating induced by meal ingestion [33], and the effects of protein-containing drinks on appetite, food consumption and gut hormones across the life span [34,35].

We believe that this Special Issue provides a comprehensive overview of the gastrointestinal mechanisms contributing to appetite and energy intake regulation. Over the last decade, there have been substantial advances in our understanding of a number of areas—from the sensing of meal content, texture and palatability, signal transmission to the brain, signal processing and the translation into behavioural responses, through to the characterisation of dysfunctions in eating-related disorders, specifically obesity and the anorexia of ageing. Hence, while classically, the brain was viewed as the centre of appetite control mechanisms, it is now clear that many of the signals that activate the pathways involved in appetite control originate in the gastrointestinal tract. The powerful effects of bariatric surgery on gut hormone release, appetite suppression and sustained weight loss attest to this. The challenge for the next decade is to translate this knowledge, particularly that relating to the cellular mechanisms underlying nutrient sensing and gut hormone release, into effective dietary and pharmaceutical approaches for both the prevention and management of eating-related disorders.
